# A New Parasitic Archamoeba Causing Systemic Granulomatous Disease in Goldfish Extends the Diversity of Pathogenic *Endolimax* spp.

**DOI:** 10.3390/ani13050935

**Published:** 2023-03-05

**Authors:** Maria Constenla, Oswaldo Palenzuela

**Affiliations:** 1 Departament de Biologia Animal, de Biologia Vegetal i Ecologia and Servei de Diagnostic Patologic en Peixos, Universitat Autonoma de Barcelona (UAB), 08193 Barcelona, Spain; 2 Instituto de Acuicultura Torre de la Sal (IATS, CSIC), 12595 Ribera de Cabanes, Spain

**Keywords:** parasites, fish, archamoebae, systemic granulomatosis, *Carassius auratus*, *Iodamoeba*

## Abstract

**Simple Summary:**

It is almost 50 years since amoeba-associated granulomatosis was first described in goldfish (*Carassius auratus*), but the aetiologic agent has never been identified. In this study, a new archamoeba species, *Endolimax carassius*, is characterised as the causative agent of systemic granulomatous disease in goldfish. Phylogenetic analyses determined the new goldfish parasite species as a genotype sister of *Endolimax piscium*, the causative agent of systemic granulomatous disease in Senegalese sole. The fish genotypes clustered with other available genotypes from mammals in a well-supported *Endolimax* clade within the Mastigamoebidae.

**Abstract:**

*Endolimax* is a genus of intestinal amoebae which stands among the least known human protists. Previous studies on amoebic systemic granulomatosis of a marine fish (*Solea senegalensis*) resulted in the unexpected characterization of a new organism which was related to *Endolimax* and named *E. piscium*. The existence of multiple reports of systemic granulomatosis caused presumptively by unidentified amoebae in goldfish lead us to investigate the organism involved in goldfish disease. Analysed goldfish presented small whitish nodules in the kidney, which correspond to chronic granulomatous inflammatory reactions with a ring-layer of amoebae in the periphery. Amoebae were amitochondriate and were located in a parasitophorous vacuole within macrophages, as previous studies on this condition in goldfish and other freshwater fish pointed out. SSU rDNA characterization confirmed a new *Endolimax* lineage which appears closely related to *E. piscium*, but the molecular evidence, distinct pathological features and lack of ecological overlapping between the hosts support their assignment to a new species, *E. carassius*. The results support the existence of a considerable unexplored diversity of *Endolimax* spp. among fish, and their proper characterization can contribute to an understanding of Archamoebae evolution and pathogenic potential.

## 1. Introduction

*Endolimax nana* is a long-known endo-commensal or parasitic archamoebae of human and non-human primates. Reports have also described *Endolimax* spp. in birds, amphibians, reptiles, and even in insects (reviewed in [1,2]). However, these descriptions were based on superficial morphological observations and under a general assumption of host specificity for the different animal lineages. Until recently, a single complete SSUrDNA sequence of a monkey isolate (*Cercocebus albigena*), obtained in pioneering molecular phylogenetic studies of Entamoebidae [3], remained the only *E. nana* reference genotype available in public repositories. However, a recent PCR survey of human faecal samples using a panel of intestinal amoeba primers detected several new SSU rDNA segments from *Endolimax nana* genotypes grouping in two phylogenetically distinct human and non-human primate lineages [4]. Further studies into *Endolimax* sp. genetic diversity have recently been published, including the release of quality Sanger reads of cloned rDNA genes from human, wastewater and swine *E. nana* SSU rDNA genotypes [5,6,7]. A high degree of intrageneric variation at the SSU rDNA locus has been found for these new *Endolimax nana* sequences, and also for the closely related genus *Iodamoeba* [8]. Both genera remain as the least well-known human parasitic protists and are largely unexplored in terms of morphology, taxonomy, genetic diversity, host specificity, and epidemiology [2].

Previous research on the characterization of a cryptic granulomatous systemic disease (GSD) in cultured Senegalese sole (*Solea senegalensis*) revealed a new amoeba species clustering with *Endolimax* genotypes in phylogenetic analyses, which was named *E. piscium* [1]. Specific studies on the ultrastructure, pathology, diagnostics, and transmission of *E. piscium* have been presented elsewhere [9,10,11]. Besides the morphological and genetic differences with terrestrial *Endolimax* sp., the most noteworthy feature of *E. piscium* is its ability to breach the intestinal barrier causing systemic disease, which manifests in sole as conspicuous muscle lumps and nodules resembling abscesses in different organs [1,9]. However, covert infections in the intestinal epithelium of asymptomatic fish have also been revealed by specific studies with in situ hybridization techniques (ISH) [10].

Amoebae can cause severe diseases in fish, mainly affecting the gills but also systemic infections in different species, as reviewed in [12]. Among these, several case reports of GSD in goldfish (*Carassius auratus*) have existed since 1977, including observations of amoebae-like organisms [13,14,15]. A retrospective review of these reports after the full characterization of *E. piscium* suggested that the causative agent could be related to *E. piscium* and the aim of this work was to search for and characterize *Endolimax*-like parasites in goldfish. A new lineage of *Endolimax* was found and characterized. Phylogenetic, morphological and pathological data demonstrate a close relationship between *E. piscium* and the goldfish lineage, but support them being independent species.

## 2. Materials and Methods

Fish and biological samples. Goldfish were obtained from commercial pet-store suppliers for the Universitat Autònoma de Barcelona (UAB) School of Veterinary Medicine practical training programs in fish diseases. They were maintained in glass tanks with individual closed recirculation filters and were subject to periodical monitoring and correction of their water physical–chemical quality parameters through partial water changes. Fish showing signs of distress or used during students practical skills training programs were sacrificed with an overdose of anaesthetic with 2-Phenoxyethanol (Panreac Applichem, Barcelona, Spain), which was progressively introduced into the water for 2 min up to a concentration of 4 mL.L^−1^. They were necropsied and examined following standard anatomopathological routines. Samples of the different organs were fixed in 10% Neutral Buffered formalin and embedded in paraffin, and sections were stained with haematoxylin and eosin for histological examination. Parallel samples were taken in 80% ethanol for retrospective studies with molecular techniques. Additional samples of kidney from three specimens were processed for transmission electron microscopy (TEM) studies. The samples were fixed in EM grade 2.5% glutaraldehyde (Merck, Darmstadt, Germany) in 0.1 M cacodylate buffer (pH 7.4, Sigma-Aldrich, Madrid, Spain), and processed as previously described [9]. Ultra-thin sections were examined using a Jeol 1400 120 KV transmission electron microscope. Measurements were taken from structures of amoeba cells observed via TEM (*n* = 7).

SSU rDNA sequencing and phylogenetic inference. Ethanol-fixed samples from goldfish kidneys with presumptive amoebiasis (as determined by the macroscopical and histopathological examination) were used for DNA extraction using a spin microcolumn and silica-based commercial kit (Roche Diagnostics). Primary amplification of amoebae SSU rDNA was carried out as described previously [1]. Briefly, subterminal primers MM18Sf and MM18Sr [16] were used in 50 uL PCR reactions containing 1× Taq buffer with 2.5 mM MgCl_2_, 0.2 mM deoxyribonucleotide triphosphate (dNTP) and 1 U High-Fidelity Taq DNA polymerase, and 25 pmol of each primer. Cycling conditions consisted of an initial denaturation (3 min 94 °C) and 35 amplification cycles (94 °C per 1 min, 55 °C per 1 min, 72 °C per 1 min) followed by a final, 8 min incubation at 72 °C. Amplification products were analysed on TAE agarose gels, and the amplicons were ligated into a plasmid vector (PCR4-TOPO, Invitrogen, Waltham, MA, USA), which was used to transform competent *E. coli*. Transformants were selected on LB-agar plates, and plasmids were purified from overnight cultures in liquid media. The presence of the expected insert’s size was confirmed by restriction digestion analysis with EcoRI enzyme. Both strands of cloned products were Sanger sequenced [17] using M13F and M13R primers, and additional walking primers s1, sx, r1 and r2 were designed from the partial reads [1]. The sequences were assembled and curated by eye inspection of the electropherograms using the software Geneious Prime v2022.2.2. The final consensus sequence was inserted in an alignment with selected Amoebozoa sequences, which was refined by eye under ARB software [18] according to secondary structure criteria. Aligned positions were sampled using different manually built masks varying in the stringency of the alignment homology criteria. The final dataset consisted of 1547 positions. The datasets were analysed for phylogenetic inference using maximum likelihood (ML) and Bayesian estimation methods. ML analyses were carried out using different algorithms as implemented in PhyML and RaxML programs [19,20] and Bayesian inference was conducted in MrBayes v3.2.6 [21]. Variations of alignment sampling masks, taxa sampling, substitution models and parameters were explored to test robustness and the stability of the reconstructions but a general time-reversible model (GTR) with a gamma-distributed variation rate (6 categories) + invariable sites (G+I) was chosen in final analyses.

qPCR diagnosis and ISH. Upon detailed inspection of the new genotype, the repertory of probes designed for *E. piscium* and routinely used for qPCR and ISH diagnostics of this species in Senegalese sole [10] were evaluated in silico and potentially cross-reacting probes were tested in vitro. SYBR-based qPCR using primers Ep459fQL18 and Ep634rQL20 was tested with a total of 29 goldfish samples procured from the UAB training programs, with selected Senegalese sole samples from the Institute of Aquaculture “Torre de la Sal” (IATS) fish parasitology diagnostics service collection and with both cloned targets. The goldfish samples processed for qPCR consisted of a portion of the posterior part of the visceral package including mostly intestinal tissue, but did not include kidney. In addition, histological sections with confirmed granulomatous lesions (mostly kidney) were selected for in situ hybridization studies. Both procedures were carried out as previously described [10].

## 3. Results

### 3.1. Morphological and Histopathological Observations

**Figure 1:**
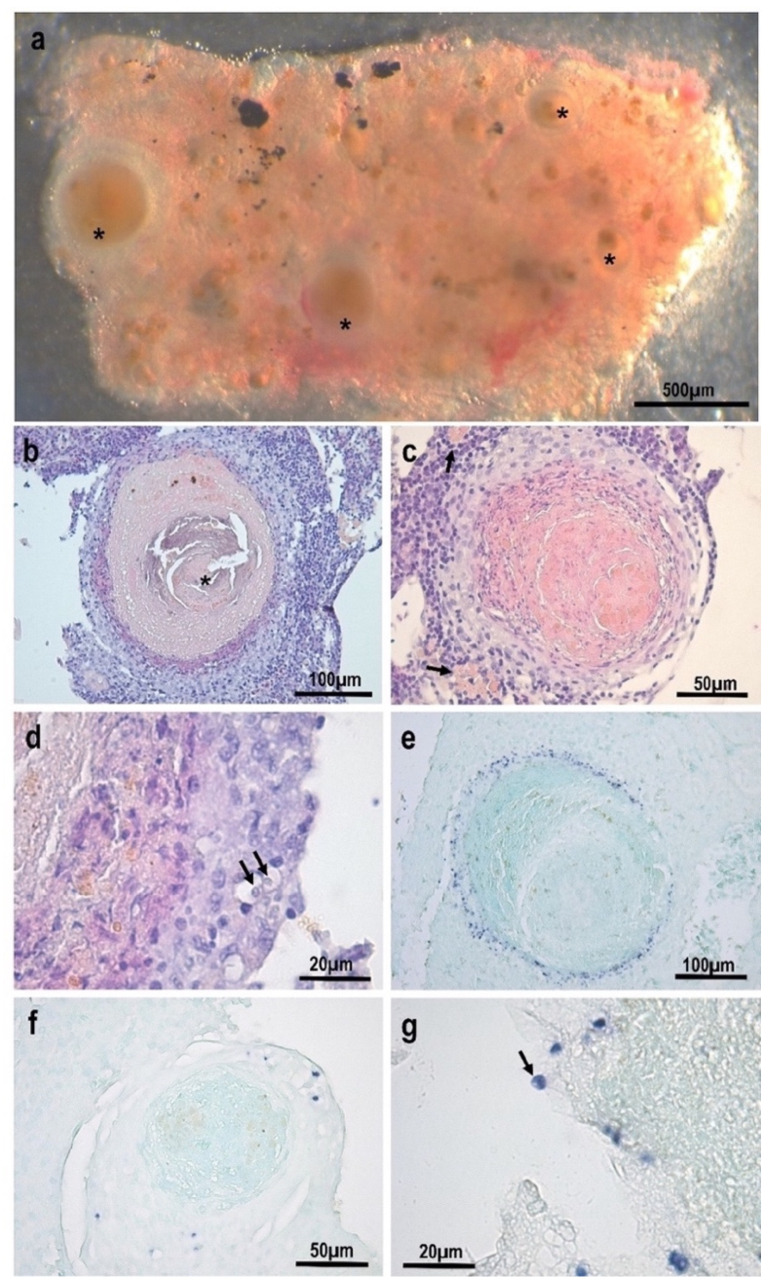
Lesions caused by *Endolimax carassius* in the kidney of goldfish (*Carassius auratus*): (**a**) macroscopic nodules (*) surrounded by a layer of clearer material; (**b**–**c**) granulomatous inflammatory reactions with a large necrotic core. Note the presence of calcified material in the core (*) and macrophage centres (arrows) at the periphery of the lesions; (**d**) detail showing two amoebae inside macrophages (arrows); (**e**–**g**) ISH-stained histological sections with amoebic lesions: parasite cells are stained purple within the inflammatory layer of the granuloma with variable, but generally low infection intensity. Some apparently extracellular parasites could also be detected outside the granuloma (arrow).

Small whitish nodules, ranging between 50 and 450 µm in diameter, were observed in the kidney of infected goldfish (Figure 1a). Histologically, the nodules consisted of chronic granulomatous inflammatory areas, with a large core of homogeneous necrotic tissue surrounded by fibroblasts and macrophages (Figure 1b,c). Calcified material was observed among necrotic material in the core of some granulomas (Figure 1b). Macrophage centres were usually observed at the periphery of the granulomas (Figure 1c). Amoebae were detected in some granulomas within macrophages, among the external inflammatory cells and fibroblasts, but generally in a very small number (Figure 1d).

In situ hybridization using *E. piscium* probes clearly labelled these amoebae with a dark purple precipitate (Figure 1e–g). The histological sections stained using ISH clearly showed that the parasites were located as a ring layer surrounding the granuloma core (Figure 1e) and they also corroborated the small number of parasite cells in most of the lesions (Figure 1f). Apparently, extracellular amoeba were also detected at the periphery of one granuloma (Figure 1g).

**Figure 2:**
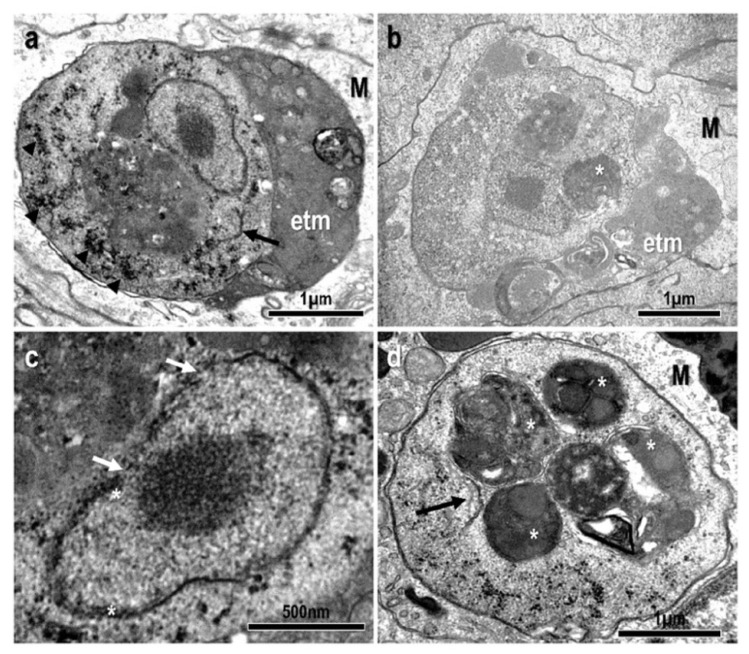
Transmission electron micrographs of parasites in *Carassius auratus* kidney. (**a**,**b**) Ultrastructure of the amoebae trophozoites, located inside parasitophorous vacuoles within host macrophages (M). Note the vesicular nucleus with central nucleolus, rough endoplasmic reticulum (arrow), small glycogen granules (arrowheads), and vacuole-like structures with degraded material (*). Large amount of expelled trophic material (etm) is adjoining the amoeba filling the space inside the parasitophorous vacuole; (**c**) detail of a nucleus with nuclear pores (arrows) and peripheral chromatin (*); (**d**) amoeba with digestive vacuoles (*) containing withered materials at different degrees of degradation, myelin figures and strips of rough endoplasmic reticulum (arrow).

At the ultrastructural level, amoebae were located inside macrophages, and surrounded by a parasitophorous vacuole. Parasite stages were mostly round in shape with an average diameter of 3.478 ± 0.379 µm. They contained one vesicular nucleus (1.265 ± 0.110 µm in diameter) displaying a large central nucleolus (diameter 0.476 ± 0.076 µm) (Figure 2a–c). The nucleus was demarcated by a double nuclear membrane with prominent pores and small areas of associated peripheral chromatin (Figure 2c). The parasites’ cytoplasm contained small glycogen granules, often forming aggregates (Figure 2a), and membranes resembling rough endoplasmic reticulum (Figure 2a,d). Other structures characteristic of amoebae such as digestive vacuoles containing particulate material or products of lysosomal action were commonly observed (Figure 2a–c). Expelled trophic materials filling the peripheral space of the parasitophorous vacuole were often present (Figure 2a,b). Neither mitochondria nor mitosomes were observed.

### 3.2. SSU rDNA Characterization and Phylogenetic Inference

Primers MM18sf and MM18sr amplified a ∼3000 bps product from infected goldfish kidney. The product obtained from a single fish was cloned and Sanger sequenced and the consensus from several reads, with 2934 bps, was deposited in Genbank with accession number ON646298. The most significant matches in Blast searches against NCBI databases (highest Bit-Score) were *Endolimax piscium* clones, with which the new genotype shared an 87.5% pairwise identity with 100% coverage. These were followed by hits with segments of *Iodamoeba* sp., *Endolimax* sp. And other available archamoebae genotypes, reaching high pairwise homology (up to 93.7%) along the matching segment located between nts. 2016–2302 of the new sequence.

**Figure 3:**
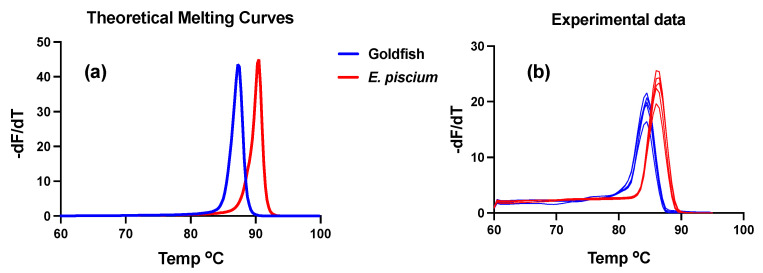
Derivative melting curves of *Endolimax piscium* and the new goldfish genotype amplified via qPCR. (**a**) The theoretical curve predicted in silico using uMelt [22] with the sequences of both genotypes; (**b**) experimental data obtained with DNA samples from amoebae-infected sole and goldfish, using the *E. piscium* qPCR test described in [10].

The *Endolimax piscium* ISH probe 1999L24 Amsolea was 100% homologous with the goldfish SSU rDNA genotype, whereas the other probe included in the same procedure (1575L24 Amsolea) presented seven mismatches. Of the qPCR primers, the forward Ep459fQL18 was 100% homologous and the reverse primer Ep634rQL20 presented a single mismatch, which suggested a high likelihood of cross-species amplification using this test. This lead us to explore the melting curves of the amplicons in both genotypes, and to predict sufficient differences in the curves to discriminate both genotypes after amplification (Figure 3a). This prediction was further verified with qPCR using clinical samples and cloned products from both genotypes. Out of 29 goldfish samples tested, 24 were PCR-positive with the *E. piscium* test (Ct cut-off value < 38) and the resulting amplicon melting curves matched the in silico predictions with minor Tm peak differences (Figure 3b).

**Figure 4:**
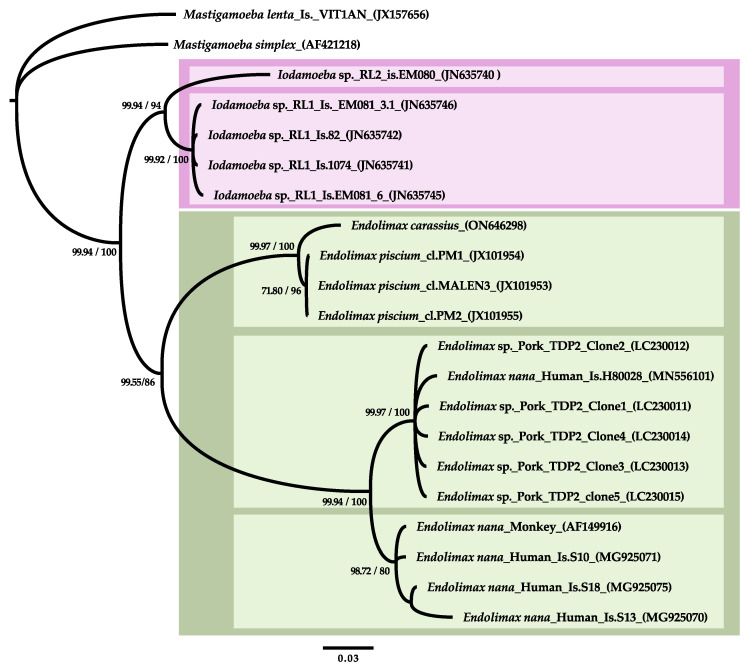
Phylogenetic analysis of *Endolimax carassius* SSU rDNA and selected representative Mastigamoebidae sequences. The analysis used 1547 alignment positions. The topology was inferred using Bayesian inference with a gamma distribution (six categories) and a proportion of invariable sites. Numbers at nodes represent the posterior probabilities followed by the bootstrap support obtained in a maximum likelihood analysis using the same dataset and model. All branches are drawn to scale. The scale bar represents 0.03 substitutions per site. Well-supported subclades are highlighted in coloured boxes.

Phylogenetic analyses resolved the new goldfish genotype sister to *Endolimax piscium*, and the fish genotypes clustered with other available genotypes from mammals in a well-supported *Endolimax* clade (Figure 4). In general, two distinct terrestrial *Endolimax* lineages were well supported although the inclusion of short sequence segments, particularly those from NGS studies from wastewater obtained in [6], resulted in the poor resolution of these OTUs. Using the longest representative quality sequences, the first lineage included swine isolates generated in a single study and *Endolimax* sp. (H80028) with a human faecal origin. The other major lineage included the reference *E. nana* AF149916 and three isolates from human stool. *Iodamoeba* sp. genotypes branched off as sister to the *Endolimax* spp. clade in our analyses. This arrangement was consistently resolved by phylogenetic inference using Bayesian methods and different implementations of maximum likelihood methods and substitution models. However, the support values for the main nodes were sensitive to taxon sampling and, particularly, to the stringency of the alignment trimming criteria.

### 3.3. Description of the Species: Endolimax carassius *n. sp.*

- Type host: *Carassius auratus*
- Locality: The parasite was detected in *Carassius auratus* from commercial ornamental fish suppliers in Barcelona, Spain. Previous reports in North America, Central Europe and Israel. Presumed to be distributed worldwide
- Location in the host: Systemic: mostly found within the inflammatory layer at the periphery of granulomatous lesions in the kidney. Detected via qPCR in samples from asymptomatic fish with a high prevalence (83%);
- Material deposited: A partial SSU rDNA (2934 bps) comprising the coding region between helixes 1–48 was deposited in GenBank with Accession no. ON646298;
- Taxonomic remarks: High morphological and pathogenic resemblance with *Endolimax piscium* but with distinct SSU rDNA genotype (87.5% pairwise identity). Phylogenetic clustering with terrestrial *Endolimax* sp. genotypes and with genotypes of the genus *Iodamoeba* as the closest sister group.

## 4. Discussion

It is almost 50 years ago since cases of GSD attributed to amoebae were first described in goldfish from North America [13]. Related cases of systemic inflammatory granulomatous lesions involving amoebae-like organisms in this host were also reported in Europe and Israel in the early 1990s [23,24,25]. However, some authors suggested a *Dermocystidium*-like organism as the etiological agent of goldfish GSD [23]. Extensive work to study this condition was conducted in [14], whose authors reported a high prevalence (59%) of compatible granulomatous lesions in goldfish from different local sources and provided a comprehensive pathological and ultrastructural description of the causative agent. However, their findings were not conclusive due to a lack of unambiguous morphological features allowing a diagnosis beyond an “amoeba-like” organisms. Similar, and possibly related pathological conditions have also been reported in other freshwater fish such as dwarf gourami (*Colisa lalia*) [26], European catfish (*Silurus glanis*) [27] and tench (*Tinca tinca*) [28] (reviewed in [12]). It was unforeseen that the causative agent of the emerging GSD syndrome in culture sole (*Solea senegalensis*) was identified as an amitochondriate Archamoebae representing a new piscine *Endolimax* clade [1]. In this work, the characterization of the parasite causing goldfish GSD was tackled from the hypothesis that it could related to *Endolimax piscium*, on the basis of noted similarities in the morphology and pathogeny of Senegalese sole and goldfish GSD.

The results of the histopathological and ultrastructural analyses in this work are largely in agreement with previous studies on this condition in goldfish and other freshwater fish. In all cases, the lesions consisted of inflammatory granulomas, most often present in kidney but also in other organs such as liver, spleen or even brain, associated with very small amoeba-like organisms which are usually present (albeit often in small numbers and difficult to detect) at the peripheral layers of inflammatory cells. This could be particularly well observed using ISH staining. The low numbers of parasites could be indicative of the chronicity of the disease [23]. There are, however, some differences in pathogeny between goldfish GSD and the lesions caused by *E. piscium* in Senegalese sole. On the one hand, macroscopic lesions are much larger and more extensive in Senegalese sole, presenting a completely liquefied core characteristic of an abscess in most of the cases [9], and contrasting with the small nodules present in goldfish (only a few millimeters) and other freshwater species (up to 10 mm in tench). In addition, large numbers of parasites are usually involved in the lesions suggesting a more acute course of development. On the other hand, while lesions in goldfish (and, seemingly, in tench [28]) seem to be more restricted to the kidney, in Senegalese sole they mainly affect skeletal muscle. Histologically, in addition to the granulomatous lesions present in both species, *E. piscium* has also been associated with extensive necrosis and diffuse inflammation in different organs. A progression of the lesions in goldfish GSD was suggested in [23], starting from the kidney (early stages of the disease) to spleen, liver, mesenteries and even to the heart (late infections). However, histopathological analyses and specific studies with *E. piscium* using molecular diagnostic techniques revealed the parasites within the intestinal epithelium in asymptomatic sole, indicating the intestinal mucosa as the main route of invasion in the pathogenesis of sole GSD, from which it can spread to other organs [10,11]. This location of *E. piscium* in the host also suggest that the parasites can remain as endocommensal or with a minimally invasive role at the intestinal epithelium. This possibility has not been confirmed in freshwater fish yet, but the detection of *Endolimax* via qPCR in a large proportion of the goldfish samples tested in this study (i.e., excluding the kidney) leaves open this possibility.

Ultrastructural observations conducted in this work are also in agreement with previous data from goldfish GSD [13,14] and they are virtually identical to those from tench [28]. The parasites were always detected intracellularly, within a parasitophorous vacuoles inside macrophages. Expelled material, probably trophic, was often present filling the peripheral space of the parasitophorous vacuole. The absence of Golgi apparatus and mitochondria in their trophozoites is also remarkable, like the absence of mitosomes or hidrogenosomes typical of amitochondriate eukaryotes. In contrast, *E. piscium* can be detected extracellularly and without trophic material outside the amoebae. In addition, dictyosome-like structures resembling Golgi apparatus and mitosomes have been described in this parasite [1]. The minute size of the trophozoites of both *Endolimax* species from fish ([1] and the present study) is also remarkable, being even smaller than *E. nana* [2].

The characterization of the SSU rDNA sequence retrieved from goldfish kidney displaying granulomatous lesions identified the causative agent as a new Archamoeba, with *Endolimax piscium* as the closest matching genotype. Both fish sequences shared 87.5% pairwise identity along their overlapping region, which covers almost the complete gene sequence. Minor (<0.1%) intraspecific genetic diversity has been found at this locus in *E. piscium* [1], but the products sequenced from goldfish in this study derived from a single fish and no heterogeneity was detected. The qPCR test routinely used for the diagnostics of *E. piscium* in sole amplified 24/29 goldfish tested and these amplicons could be consistently discriminated in the melting curves from both hosts suggesting that the SSU rDNA in the goldfish parasite presents limited variability as well. It should be highlighted that this cross-reaction will facilitate the diagnosis of goldfish GSD and discrimination from *E. piscium*, since published tests for the later species [10] can be used directly or with little modification. Considering the high prevalence of this parasite in goldfish (more than 80% in the present work) and its worldwide distribution, having effective diagnostic tools is especially relevant for the experimental use of SPF goldfish.

Although large variability has been found in the SSU rDNA sequences of terrestrial *Endolimax* and *Iodamoeba* in the current and in previous studies [4,7,8]; the biological characterization of the source isolates is nearly non-existent and the possibility that they correspond to hypervariable intraspecific genotypes vs. distinct phylogeographical or phyloecological lineages is currently unknown. In this work, the overall phylogenetic reconstruction of *Endolimax* and *Iodamoeba* main lineages within the Mastigamoebidae was in agreement with previous studies [1,29]. However, we obtained good support for a monophyletic *Endolimax* clade including terrestrial and piscine genotypes, which contrasts with a recent study using a very similar dataset [7]. We found that some of the support values were vulnerable to taxon sampling and, particularly, to the stringency of the alignment trimming criteria due to the existence of large expansion and hypervariable regions in the SSU rDNA sequences studied, which add uncertainty to the alignment even while using secondary structure criteria. In our final dataset, we included 1547 positions as the maximum reasonable number with a rather relaxed stringency criteria, while in previous studies we used mask variations between 1350 and 1493 positions with a similar dataset [1]. This range is much lower than the 2067 positions used in [7], and these differences can result in variable levels of signal to noise ratio affecting the support for certain reconstructions [30,31,32]. It is clear that piscine and terrestrial *Endolimax* genotypes have a long evolutionary history as separate subclades and their relations with *Iodamoeba* can be difficult to settle with the currently available genetic datasets.

While some of the differences discussed above in the pathogenesis of GSD in sole compared to goldfish and tench could be attributed in part to differences in the hosts responses modulating parasite virulence, tissue distribution patterns and chronicity of the lesions, the combined evidence of: (i) distinct anatomopathological and ultrastructural findings; (ii) lack of ecological overlapping between goldfish and sole; and (iii) substantial genetic differences between both isolates; support them being different species and leads us to propose *Endolimax carassius* n. sp. as the causative agent of goldfish GSD.

Members of Archamoeba are generally not well studied, probably due to their limited clinical importance and the difficulties associated with their culture in the laboratory [3] and with the amplification and sequencing of their ribosomal genes [7]. The genus *Entamoeba* is by far the most studied, mainly due to *E. histolytica*, the only true pathogenic species for humans. *E. histolytica* infections usually include an intestinal phase, asymptomatic or with severe invasive infections, and an extraintestinal phase that generally affects the liver, with possible progression to other organs such as the lungs, brain or heart through blood dissemination [33]. However, species of the genus *Endolimax* or *Iodamoeba* from terrestrial hosts are restricted to the intestinal lumen, playing an endocommensal role. In this sense, the pathogenesis of fish *Endolimax* infections presents remarkable similarities with that of *E. histolytica* and extends the diversity of pathogenic archamoebae species of concern in veterinary medicine. Considering the overwhelming ecological and phylogenetic diversity of the hosts, systemic *Endolimax* in fish are likely a diverse and cryptic group of parasites with serious pathological potential, which has emerged so far only in a small number of captive-bred hosts. It is also noteworthy that most, if not all, cases of amoebic GSD reported in fish were detected in animals reared under closed water re-circulation systems. As it has already been suggested in sole, these conditions likely favour the survival and permanence of amoebae stages outside the host and the continuous exposure to an oral-faecal contagion route, facilitating the emergence of clinic GSD due to *Endolimax* spp., particularly in facilities with intensive culture conditions [12]. The current trends of production scale-up and diversification of aquaculture species may be threatened by emerging parasitism due to *Endolimax*.

## 5. Conclusions

The characterization of *Endolimax carassius* clarifies a long-standing debate on the aetiology of goldfish GSD and extends the diversity of pathogenic *Endolimax* spp. Existing diagnostic tests for *E. piscium* can be used for *E. carassius* directly or with minimum modifications, and both genotypes can be discriminated using amplicon melting curves. Given the high prevalence of *E. carassius* observed in this work and in previous reports, the use of these tests is particularly relevant in goldfish destined for certain experimental studies, in which pathogen free or specific pathogen free status may be of the utmost importance. The results of this study support the existence of a diverse clade of *Endolimax* spp. in fish, which can have high pathogenic potential and clinical relevance in both freshwater and marine aquaculture production.

## Data Availability

Partial SSU rDNA sequence of *Endolimax carassius* (2934 bps) was deposited in GenBank with accession number ON646298.
